# *Schistosoma mansoni* Infections, Undernutrition and Anaemia among Primary Schoolchildren in Two Onshore Villages in Rorya District, North-Western Tanzania

**DOI:** 10.1371/journal.pone.0167122

**Published:** 2016-12-09

**Authors:** David Zadock Munisi, Joram Buza, Emmanuel A. Mpolya, Safari M. Kinung’hi

**Affiliations:** 1 Department of Global Health and Bio-Medical Sciences, School of Life Sciences and Bioengineering, Nelson Mandela African Institution of Science and Technology, Arusha, Tanzania; 2 Department of Bio-Medical Sciences, School of Medicine and Dentistry, College of Health Sciences, University of Dodoma, Dodoma, Tanzania; 3 National Institute for Medical Research (NIMR), Mwanza Research Centre, Isamilo Road, Mwanza, Tanzania; Centers for Disease Control and Prevention, UNITED STATES

## Abstract

**Background:**

Undernutrition and anaemia remains to be a major public health problem in many developing countries, where they mostly affect children. Intestinal parasitic infections are known to affect both growth and haemoglobin levels. Much has been reported on the impact of geohelminths on anaemia and undernutrition, leaving that of *Schistosoma mansoni* not well studied. Therefore this study intended to determine the association between *S*.*mansoni* infections, anaemia and undernutrition among schoolchildren in Rorya district, Northwestern Tanzania.

**Methodology:**

A cross-sectional study was carried among schoolchildren in two onshore villages namely Busanga and Kibuyi in Rorya district. Single stool specimens were collected from 513 randomly selected schoolchildren and processed for microscopic examination using the Kato-Katz method. Nutritional status was determined by anthropometry. Blood samples were also collected and examined for malaria parasites and haemoglobin levels using the Giemsa stain and HaemoCue methods, respectively. A pretested questionnaire was used to collect socio-demographic data and associated factors.

**Results:**

The prevalence of *S*. *mansoni* infection and malaria was 84.02% and 9.16%, respectively. Other parasites found were *Ascaris lumbricoides* (1.36%) and Hookworm (1.36%). The prevalence of stunting and wasting was 38.21% and 14.42%, respectively. The prevalence of anaemia was 29.43%, whereby 0.58% had severe anaemia. *S*. *mansoni* infection was not found to be associated with undernutrition or anaemia (p>0.05). The risk of stunting and wasting increased with increasing age (*p<*0.001). Anaemia was associated with age, sex and village of residence (p<0.05).

**Conclusions:**

*S*.*mansoni*, undernutrition and anaemia are highly prevalent in the study area. The observed rates of undernutrition and anaemia were seen not to be associated with *S*.*mansoni* infection suggesting possibly being a result of poor dietary nutrients. This study suggests that policy makers should consider Rorya district for inclusion into national schistosomiasis control and school feeding programmes.

## Introduction

Undernutrition and anaemia are still public health problems in many developing countries where they are known to mostly affect children. The two are known to affect physical and mental development as well as immunity thereby rendering the already vulnerable group more susceptible to infections with other commonly occurring bacterial and viral pathogens [1,2,3]. It is estimated that about one-fourth of African primary schoolchildren lie under the fifth percentile of United States National Center for Health Statistics (US-NCHS) reference for Height-for-Age Z-score (HAZ) and nearly 40% of pre-schoolchildren living in developing regions are anaemic [4,5].

In Tanzania, undernutrition and anaemia among schoolchildren are still a major public health problem. It has been reported that up to two-third of children are anaemic [6] and about 42.3% of schoolchildren are undernourished [7]. While factors that affect growth and development in pre-school children have been well elucidated, a lot remains to be done with schoolchildren where risk factors for anaemia and under nutrition are not well understood [8].

Besides other morbidities, intestinal parasitic infections are known to affect both the growth of children and their haemoglobin levels [9]. It has further been reported that, school age children is the group that is mostly affected by intestinal parasites and also suffers the greatest morbidity attributable to these parasites [10,11]. However, many studies that tried to examine the relationship between parasitic infections, undernutrition and anaemia paid much attention to geohelminths, leaving *S*. *mansoni* not well studied [12,13,14]. There have been very limited studies on the impact of Schistosome infections on anaemia and undernutrition [8,15,16,17,18]. Further, the few available studies have produced contradicting findings. Understanding the association between *S*. *mansoni* infection and anaemia and undernutrition will be helpful in the formulation of comprehensive interventions which aims at reducing the burden of anaemia and undernutrition in the study area and elsewhere. Therefore this study intended to determine the association between *S*. *mansoni* infections and anaemia and undernutrition among primary schoolchildren in the study area.

## Methods

### Study area

**Fig 1:**
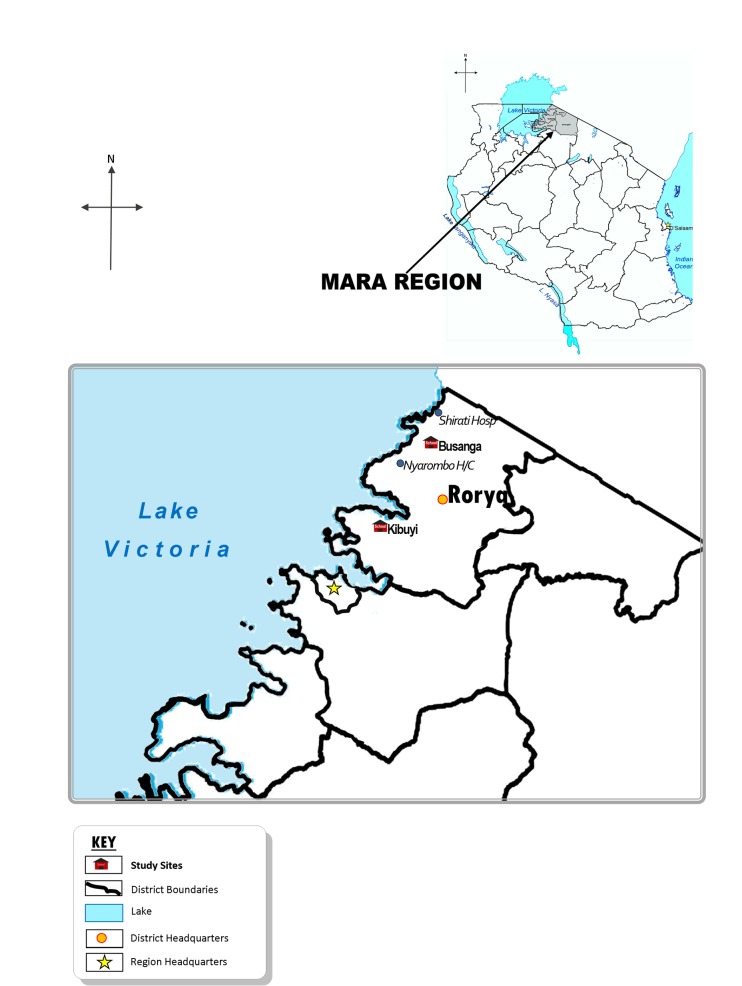
A Map of Rorya District Showing the Study Sites.

This study was conducted in Rorya district, North-western Tanzania. Rorya district is one among seven districts in Mara region. The district is bordered by Tarime district to the east, Butiama district to the south, Lake Victoria to the west and the Republic of Kenya to the North (Fig 1) [19]. The majority of inhabitants of Rorya District are from the Luo tribe. Other ethnic groups are Kurya, Kine, Simbiti, Sweta and Suba. The District is situated in the North of Tanzania and lies between latitudes 1°00"– 1°45" south of the Equator and longitudes 33° 30"– 35° 0" east of Greenwich Meridian. Rorya district has two agro-ecological zones namely the midlands and the low lands. The zones are situated between approximately attitudes 800m and 1200m with temperatures varying from 14°C to 30°C. The annual rainfall ranges from 700mm to 1200mm. The district has a total area of 9,345 square kilometers. The top five most commonly reported causes of morbidity and mortality are Malaria, Acute Respiratory Infections, Diarrhoea, Intestinal worms and Pneumonia [20].

### Study design

The current study was a cross-sectional baseline survey which formed part of a longitudinal randomized intervention trial with a registration number PACTR201601001416338 registered on the Pan African Clinical Trial Registry. The longitudinal randomized intervention trial aimed to assess the efficacy of Praziquantel treatment regimen on parasitological (egg reduction rate and cure rates) and morbidity indicators. This cross-sectional baseline survey assessed the prevalence and intensity of *S*. *mansoni* infection, nutritional status and haemoglobin levels of schoolchildren. The study also assessed the socioeconomic characteristics of parents of schoolchildren in the selected villages.

### Study population, inclusion and exclusion criteria

The study population consisted of primary schoolchildren aged 6–16 years attending primary schools in two villages of Busanga and Kibuyi in Rorya district. All schoolchildren aged 6–16 years who agreed to participate in the study and whose parents gave written informed consent were eligible for inclusion into the study. Schoolchildren who had a history of being clinically ill during the time of recruitment or had used anthelmintic drugs within a period of 6 months before the study and those whose parents refused to sign a written informed consent form were excluded from the study.

### Sample size determination and sampling procedures

This study formed the baseline survey of a longitudinal intervention trial which aimed at comparing parasitological cure rates of two different treatment regimens of praziquantel for the treatment of intestinal schistosomiasis. Therefore the sample size was calculated using a formula for comparison of two rates [21]. In the calculations we used the parasitological cure rates of praziquantel against intestinal schistosomiasis reported from a study of communities living along the shores of Lake Albert in Uganda, which reported cure rates of 41.9% and 69.1% for the single dose and two doses treatment regimens, respectively [22]. We set the level of significance at 95% and power of 90%. Adding 30% annual loss to follow-up, a total sample size of 257 schoolchildren was required per treatment group. However, we were able to recruit 256 schoolchildren for the single dose treatment group and 257 for the multiple dose treatment group or a total of 513 schoolchildren for the whole study.

Conveniently, two schools along the Lake Victoria shore were selected from two villages (Busanga and Kibuyi). A total of 246 and 267 schoolchildren were recruited from Busanga and Kibuyi primary schools, respectively. We sampled children from the preparatory to the sixth grade. Children in grade seven were excluded because they were about to do their final national examinations and they would not be around during the follow-up surveys. The number of schoolchildren selected from each grade was determined by the probability proportional to number of children in the grade. We attempted to sample equal numbers of boys and girls from each grade of which half were to be boys and half girls. Systematic random sampling method was used to obtain study participants for each sex from each grade. The schoolchildren in each grade were requested to stand in two lines, one for boys and the other one for girls and they were counted. The sampling interval was obtained by dividing the total number of each sex in the grade with the number of each sex to be investigated from that grade (N/n). After obtaining a starting point from a table of random numbers, children were sampled according to the sampling interval. The same interval was kept until the required number of children for each sex in each grade was obtained.

### Data collection

#### Assessment for demographic characteristics and risk factors for *S*. *mansoni* infection

A pre-tested Kiswahili translated semi-structured questionnaire was used to gather demographic information and risk factors for *S*. *mansoni* infection. Variables such as age, sex, socio-demographic characteristics, economic activities of parents/guardians, were assessed as potential risk factors for *S*. *mansoni* infection, anaemia and undernutrition. The questionnaire was initially developed in English and then translated to Kiswahili and back-translated to English by a different person who was blinded to the original questionnaire.

#### Stool sample collection, processing and examination

A day before stool sample collection, the study objectives were explained to the schoolteachers and children. Then schoolchildren were provided with informed consent forms to take home to their parents/guardians. They were instructed to tell their parents/guardians to read and understand the consent forms and then sign if they agree for their children to participate in the study. The next morning, children with signed informed consent forms were provided with stool containers and clean wooden applicator sticks. They were requested to bring sizeable stool samples of their own. A single stool sample was collected from each study participant. Four Kato-Katz thick smears were prepared from different parts of the single stool sample using a template of 41.7 mg (Vestergaard Frandsen, Lausanne, Switzerland), following a standard protocol [23,24,25]. Examination of Kato smears for hookworm eggs was performed within 1 hour of slide preparation. Then the Kato smears were arranged in wooden slide boxes, packed together in large container boxes and transported using the project vehicle to the laboratory of the National Institute for Medical Research (NIMR), Mwanza centre where they were preserved at room temperature. The Kato smears were examined for *S*. *mansoni* eggs by two experienced laboratory technicians one week after preparation. All Kato smears prepared for each child were used to determine *S*. *mansoni* eggs per gram of faeces (EPG) for that child. For quality assurance, a random sample of 10% of the negative and positive Kato-Katz thick smears were re-examined by a third technician. Since a template delivering 41.7mg of stool was used to prepare Kato-Katz slides, the eggs of each parasite in the slide was counted and the number of eggs was multiplied by a factor of 24 to calculate EPG for *S*.*mansoni* infection. The intensity of *S*. *mansoni* infection was calculated based on the intensity classes set by WHO as light (1–99 epg), moderate (100–399 epg) and heavy (≥ 400 epg) [25].

#### Anthropometric measurements

The children’s heights were measured using a portable stadiometer and weight was measured using a digital weighing scale. The children’s barefoot stature was recorded to the nearest 0.1 cm. Weight measures were taken to the nearest 0.1 kg without shoes and with minimum clothing. The resulting height and weight measurements were compared to a standard population of the same age group to calculate height-for-age z scores and BMI-for-age z scores. These anthropometric indices were calculated using the new World Health Organization Child Growth Standards [26]. Any child with height-for-age z scores (HAZ) and BMI-for- age z scores (BMIAZ) below or equal to -2 standard deviation (≤ -2SD) was classified as stunted and wasted, respectively. Children with HAZ and BMIAZ below or equal to -3 standard deviation (≤ -3 SD) was classified as severely stunted and severely wasted, respectively. Body mass index (BMI) was used as the index of choice for the assessment of recent under-nutrition because of its being recommended for use in both adults and adolescents [27]. As part of data quality assurance, in addition to test-retest and inter-rater reliability assessments, all anthropometric measurements were taken with calibrated and validated instruments.

Age of each participant was collected from school records as reported by parents/guardians during school registration of the children. The age was reported in years in the registration, so the midpoint of the year of birth was used, and the 15^th^ day of the month was used.

#### Determination of hemoglobin levels

Blood samples were collected by finger prick method using disposable lancets, and a sample of blood (about 100μl) was collected and used to measures venous Haemoglobin (Hb), in a HaemoCue photometer (HemoCue, Angelholm, Sweden) [28]. Children with Hb levels ≥11g/dL were considered normal. Anaemia was defined as Hb levels <11g/dL while Hb levels of <7g/dL, 7.0‐9.9g/dL and 10.0‐10.9g/dL were classified as severe anaemia, moderate anaemic and mild anaemia, respectively [29].

#### Examination for malaria parasites

After a finger prick and assessment for Hb, a thick blood smears was prepared for malaria parasites examination using the Giemsa stain method [30]. The thick blood smears were examined for malaria parasite at 100X magnification.

#### Data analysis

The collected data were entered into a database using EpiData version 3.1. Data analysis was done using STATA version 12.1 (STATA Corp, Texas, USA). Simple frequency and percentages were used in the descriptive analysis. The Chi-square test and Fisher exact test were used to compare proportions and to test for associations between prevalence of *S*. *mansoni* infection, anaemia, stunting and wasting and exposure variables as appropriate. Parasite counts were normalized by log transformation, averaged and then back transformed to the original scale. *S*.*mansoni* infection intensities were calculated as geometric mean of eggs per gram of faeces. Logistic regression analysis was performed to determine the independent effect of the independent variables with dependent variable by calculating the strength of the association between anaemia, stunting and wasting and determinant factors using odds ratio (OR) and 95% confidence intervals (CIs). Crude OR and adjusted OR were estimated by bivariate and multivariate logistic regression analysis with respective 95% CIs. Multivariate logistic regression analysis was conducted by fitting a logistic regression model. All variables with a *p*-value <0.2 in the bivariate analysis were included in the model. A *p*-value of less than 0.05 was considered as statistically significant.

#### Ethical statement

The study was approved by the Medical Research Coordination Committee (MRCC) of the National Institute for Medical Research (NIMR), Tanzania (Reference No. NIMR/HQ/R.8a/Vol. IX/1990). The study received further clearance from the District Executive Director, District Education Officer and District Medical Officer of the Rorya district council. Before commencement of the study, the research team conducted meetings with the village executive officers, teachers and pupils of selected villages and schools, respectively. During these meetings, the objectives of the study, the study procedures to be followed, samples to be taken, study benefits and potential risks and discomforts were explained. Informed consent for all children who participated in the study was sought from parents and legal guardians by signing an informed consent form. Assent was sought from children who were also informed of their right to refuse to participate in the study and to withdraw from the study at any time point during the study. After baseline, all children were given a standard dose of praziquantel (40mg/kg) and albendazole (400mg) as a single dose on separate days. Treatment with praziquantel was given after a meal which was prepared and offered at the school to minimize potential side effects. Treatment was performed immediately after baseline data collection and was done under direct observation (DOT) by a qualified nurse.

## Results

A total of 513 children between 6–16 years of age were recruited into the study. Out of these, 255 (49.71%) were males and 258 (50.29%) were females. Most of the participants (40.94%) belonged to the age group of 10–12. The majority of parents in the villages had only primary school education and about a half (49.39%) were fishermen (Table 1). The overall prevalence of stunting and wasting was 38.21% (196/513) and 14.42% (74/513), respectively. The overall prevalence of anaemia was 29.43% (151/513), with the prevalence of mild, moderate and severe anaemia being 19.69%, 9.16% and 0.58%, respectively (Table 2). The overall prevalence of *S*.*mansoni* infection was 84.02% (431/513). The overall prevalence of malaria was 9.16% (47/513) with more than 90% (43/47) of the malaria positive children being at Busanga primary school. The prevalence for *A*. *lumbricoides* and hookworm infections was 1.36% (7/513) and 1.36% (7/513), respectively. The Geometric mean egg counts per gram of faeces (epg) for S.*mansoni* was 167.13 (95% CI: 147.19–189.79), with the minimum and maximum eggs per gram of faeces being 6 and 8,496 epg respectively. The distribution of infection intensity was light (28.65%), moderate (33.53%) and heavy (21.83%) of the study participants.

**Table 1:** Socio-Demographic Information of Schoolchildren who Participated in the Study by Village.

Characteristic	Village		Total n (%)	*p-Value*
	Busanga n (%)	Kibuyi n (%)		
**Sex (n = 513)**				
Male	121 (49.19)	134 (50.19)	255 (49.71)	0.821*
Female	125 (50.81)	133 (49.81)	258 (50.29)	
**Age (in years) (n = 513)**				
6–9	87 (35.37)	68 (25.47)	155 (30.21)	0.037*
10–12	97 (39.43)	113 (42.32)	210 (40.94)	
13–16	62 (25.20)	86 (32.21)	148 (28.85)	
**Parent is a farmer (n = 488)**				
No	139 (58.40)	128 (51.20)	267 (54.71)	0.110*
Yes	99 (41.60)	122 (48.80)	221 (45.29)	
**Parent is doing businesses (n = 488)**				
No	198 (83.19)	220 (88.00)	418 (85.66)	0.130*
Yes	40 (16.81)	30 (12.00)	70 (14.34)	
**Parent’s level of education (n = 488)**				
No formal education	25 (10.50)	23 (9.20)	48 (9.84)	0.075**
Primary education	153 (64.29)	184 (73.60)	337 (69.06)	
Secondary education	38 (15.97)	20 (8.00)	58 (11.89)	
University/Collage education	3 (1.26)	3 (1.20)	6 (1.23)	
Don’t know	19 (7.98)	20 (8.00)	39 (7.99)	
**Parent is fishing (n = 488)**				
No	125 (52.52)	122 (48.80)	247 (50.61)	0.411*
Yes	113 (47.48)	128 (51.20)	241 (49.39)	

*p*-values

*Chi-square statistic

**Fisher exact test

**Table 2:** Prevalence of *S*. *mansoni* Infection, Malaria, Anaemia and Undernutrition by Village (n = 513).

Characteristic	Village		Total n (%)	*p-Value*
	Kibuyi n (%)	Busanga n(%)		
**Stunting**				
Normal	151 (56.55)	166 (67.48)	317 (61.79)	0.037*
Moderate stunting	91 (34.08)	61 (24.80)	152 (29.63)	
Severe stunting	25 (9.36)	19 (7.72)	44 (8.58)	
**Wasting**				
Normal	221 (82.77)	218 (88.62)	439 (85.58)	0.113**
Moderate wasting	35 (13.11)	24 (9.76)	59 (11.50)	
Severe wasting	11 (4.12)	4 (1.63)	15 (2.92)	
**Anaemia**				
Normal	154 (57.68)	208 (84.55)	362 (70.57)	<0.001**
Mild anaemia	69 (25.84)	32 (13.01)	101 (19.69)	
Moderate anaemia	43 (16.10)	4 (1.63)	47 (9.16)	
Severe anaemia	1 (0.37)	2 (0.81)	3 (0.58)	
**S.mansoni infection**				
Negative	56 (20.97)	26 (10.57)	82 (15.98)	<0.001*
Positive	211 (79.03)	220 (89.43)	431 (84.02)	
**Malaria infection**				
Negative	263 (98.50)	203 (82.52)	466 (90.84)	<0.001**
Positive	4 (1.50)	43 (17.48 |)	47 (9.16)	

*p*-values

*Chi-square statistic

**Fisher exact test

### The association between *S*. *mansoni* infection and stunting

Bivariate logistic regression analysis showed that, stunting was not associated with *S*.*mansoni* infection (*p*>0.05). However, it was significantly associated with age of children and the village in which the children lived. Accordingly children within 10–12 years range had 6.6 times higher odds of being stunted as compared to children aged 6–9 years (*p<*0.001). Likewise, children aged 13–16 years had 16.25 times higher odds of being stunted as compared to children aged 6–9 years (*p<*0.001). Children at Kibuyi village had 1.59 times odds of being stunted as compared to children at Busanga Villages (p = 0.011). Children of farmers had 1.66 times higher odds of stunting as compared to those whose parents were not farming (*p* = 0.007). Multivariate logistic regression analysis was conducted to fit a model including all variables with a *p*-value of ≤0.2 in the bivariate analysis for stunting. Therefore, age group of the study participants, village, reporting parent farming and history of having bloody diarrhoea in the past two weeks were included in the model for analysis. Controlling for other factors, age was the best predictor of stunting among school-age children (Table 3).

**Table 3:** Multivariate Logistic Regression Analysis for Factors Associated With Stunting amongst School Children at Busanga and Kibuyi Villages, Rorya District, North-Western Tanzania.

Risk factors	Categories	Adjusted OR (95% CI)	*p*-value
**Sex (n = 513)**	Male	1	
	Female	1.09 (0.73–1.64)	0.670
**Age (in years) (n = 513)**	6–9	1	
	10–12	5.41 (2.89–10.14)	<0.001
	13–16	14.09 (7.30–27.17)	<0.001
**Village (n = 513)**	Kibuyi	1	
	Busanga	0.76 (0.50–1.16)	0.199
**Malaria infection (n = 513)**	Negative	1	
	Positive	0.67 (0.30–1.51)	0.342
**Parent is a farmer (n = 488)**	No	1	
	Yes	1.33 (0.89–2.01)	0.164
**Had bloody diarrhoea during the past two weeks (n = 488)**	No	1	
	Yes	0 .77 (0.38–1.57)	0.466

### The association between *S*. *mansoni* infection and wasting

On bivariate logistic regression analysis, wasting was observed not to be associated with *S*. *mansoni* infection (p>0.05). However it was significantly associated with age and history of having stomach ache during the past two weeks (p<0.05). Controlling for other factors on multivariate logistic regression analysis, the AOR indicated that age was the best predictor of wasting among schoolchildren in the study area (Table 4).

**Table 4:** Multivariate Logistic Regression Analysis of Factors Associated with Wasting amongst Schoolchildren at Busanga and Kibuyi Villages, Rorya District, North-Western Tanzania.

Risk factors	Category	Adjusted OR (95% CI)	*p*-value
**Sex (n = 513)**	Male	1	
	Female	0.94 (0.55–1.61)	0.832
**Age (in years) (n = 513)**	6–9	1	
	10–12	2.01 (0.95–4.66)	0.068
	13–16	4.21 (1.94–9.17)	<0.001
**Village (n = 513)**	Kibuyi	1	
	Busanga	0.75 (0.44–1.30)	0.312
**Parent is a farmer (n = 488)**	No	1	
	Yes	0.57 (0 .32–1.00)	0.052
**Parent is doing businesses (n = 488)**	No	1	
	Yes	1.52 (0.59–2.48)	0.593
***Schistosoma mansoni* infection (n = 513)**	Negative	1	
	Positive	0.59 (0 .30–1.14)	0.117
**Stomach pain in the past two weeks (n = 488)**	No	1	
	Yes	0.66 (0 .39–1.12)	0.120

### The association between *S*. *mansoni* infection and anaemia

Bivariate logistic regression analysis showed that anaemia was not associated with *S*. *mansoni* infection (*p*>0.05). However, it was significantly associated with sex of the children, age, village in which children lived and whether the children reported their parents doing business or not. Multivariate logistic regression analysis was conducted to fit a model including all variables with a *p*-value of ≤0.2 in the bivariate analysis for anaemia. Sex, age group of the study participants, village of residence, parent doing business, parent’s level of education and *S*. *mansoni* infection status were included in the model for analysis. Controlling for other factors, sex, age and village of residence were the best predictor of anaemia among school children in the study area (Table 5).

**Table 5:** Multivariate Logistic Regression Analysis of Factors Associated With Anaemia amongst Schoolchildren at Busanga and Kibuyi Villages, Rorya District, North-Western Tanzania.

Risk factors	Category	Adjusted OR(95% CI)	*p*-value
**Sex (n = 513)**	Male	1	
	Female	1.87 (1.15–3.05)	0.012
**Age (in years)**			
	13–16	1	
	10–12	4.78 (2.35–9.73)	<0.001
	6–9	28.24 (12.24–65.11)	<0.001
**Village (n = 513)**			
	Busanga	1	
	Kibuyi	8.77 (4.81–16.00)	<0.001
**Malaria infection (n = 513)**			
	Negative	1	
	Positive	0.93 (0.36–2.38)	0.880
**Parent’s level of education (n = 488)**			
	No formal education	0.47 (0.16–1.38)	0.170
	Primary education	0.78 (0.36–1.69)	0.536
	Secondary education	1	
	University/Collage education	1.78 (0.20–15.79)	0.603
	Don’t know	1.40 (0.48–4.06)	0.537
**Parent is doing businesses (n = 488)**			
	No	1	
	Yes	0.54 (0.25–1.16)	0.113
***Schistosoma mansoni* infection (n = 513)**			
	Negative	1	
	Positive	1.09 (0.55–2.18)	0.800

## Discussion

Undernutrition and anaemia have continued to be major public health problems in many developing countries [4]. The two mainly affect school-aged children who are also the victims of parasitic infections. Studies have indicated that infections with parasites may exacerbate nutritional deficiency thereby greatly affecting their physical and intellectual development [10,11]. In the present study we investigated the association between *S*.*mansoni* infection, undernutrition and anaemia among schoolchildren in two onshore villages in Rorya district, North-western Tanzania.

The study found the prevalence of stunting and wasting to be high as categorised by WHO classification of severity of malnutrition by prevalence ranges [31]. Though this study did not find any association between undernutrition and *S*.*mansoni* infection, the prevalence of *S*.*mansoni* in the study area was very high (84.02%). However, both stunting and wasting were significantly associated with age, whereby older children were more stunted and wasted than younger children suggesting chronic nutritional insult other than intestinal helminth infection. However, recently there has been increased recognition of chronic intestinal protozoa infections as the cause of malnutrition in children and have been proposed for consideration as neglected tropical diseases that cause significant morbidity in children [32,33]. Therefore, besides the possibility of chronic inadequate dietary nutrients, chronic intestinal protozoa infections may account for the observed rate of malnutrition. The prevalence of stunting in this study is slightly lower compared to a prevalence of 42.3% which was reported in same district, Northern Tanzania and 42.7% reported in Mpwapwa district, Central Tanzania [7,34]. This observed difference is likely to be due to differences in climatic conditions between our study site which lies along the shores of Lake Victoria and the other two sites which are semi arid and endure regular food shortages [35,36]. Adverse climatic conditions are known to affect food security and increase the risk of infectious diseases [37]. The observed prevalence of wasting in this study was slightly higher than what has been reported in Same district (11.7%), but it was lower than what has been reported in Mpwapwa district (34.7%) [34]. This observed difference is likely to be due to fluctuations observed on this nutritional indicator. Wasting is an indicator of acute nutritional shortage and is therefore subject to spatial and temporal fluctuations reflecting acute nutritional insult. In addition, study methodologies used by the two studies and socio-economic differences may account for the observed differences on the prevalence of wasting. Further, the present study reports a prevalence of anaemia among study participants of 29.43%. This prevalence is high and classified as a moderate public health problem according to the WHO Classification of anaemia [4]. This observed prevalence of anaemia is lower as compared to a previously reported prevalence of 62.4% in the lake zone by Lwambo *et al* [38]. This observed difference in the prevalence of anaemia could be attributed to the changing patterns in prevalence of anaemia in the region as a result of changing epidemiological patterns of parasitic infections. This study also reports very low prevalence of hookworm and *Ascaris lumbricoides*.

Our study found that stunting was not associated with *S*.*mansoni* infection, a finding which has also been reported by Mekonnen *et al* in Ethiopia [8]. This observation suggests that *S*. *mansoni* infection is not an important factor in the aetiology of stunting in this area. The present study also found that age was a significant predictor of both stunting and wasting, with older children having highest chance of being stunted or wasted as compared to the youngest. It has been reported that with maturity, children’s household socio-economic characteristics may act in conjunction with behavioural and biological variables as important risk factors for nutritional status [39]. In addition, older children tend to be more active and lose a greater amount of energy while playing. The excess energy loss in combination with inadequate dietary nutrients could make them undernourished [40]. Wasting seemed to be more common among children who reported their parents not to be involved in farming which is likely to be due to the fact that households with farming parents are more likely to be food-secure as opposed to households with non farming parents.

Sex differences were not observed for both stunting and wasting, a finding similar to what was reported by Herrador [41], suggesting equal risk exposure for both boys and girls. However, other studies have reported that boys were more wasted and stunted than girls [40,42] citing biological factors, inequalities in resource allocation within households and socio-cultural factors to be the likely cause of the observed difference in the risk of undernutrition between boys and girls [40].

Anaemia is known to be a major public health problem particularly among schoolchildren in Tanzania with the most common type being nutritional as a result of inadequate dietary intake [43,44]. The prevalence of anaemia among school aged children in the current study (29.43%) is of moderate public health problem according to WHO classification [4]. This reported prevalence is higher compared to what has been reported in the nearby district of Sengerema (19.5%) but lower to what was reported in Kilosa district, Central Tanzania (43.4%) [45,46]. This observed difference could be due to differences in the age of study participants, climatic conditions of the study areas which may affect food security as already reported [37] and the difference in the prevalence of malaria which is known to greatly impact on haemoglobin levels [47]. In the current study, anaemia was most prevalent among schoolchildren in the village with low *S*.*mansoni* and malaria prevalence suggesting that anaemia among schoolchildren in the study area was most likely to be the result of dietary deficiency and probably other causes.

The study further observed that girls were more likely to be anaemic compared to boys a finding which has also been reported elsewhere [48,49,50]. This observation is likely to be due to unhealthy diet among girls and regular menstruation among older school girls [48,50]. This study noted that age is an important predictor of anaemia and being at a younger age carried a higher risk of being anaemic as compared to being older, the same observation has been reported by other studies [48,51]. This observation suggests that children are admitted to school while already anaemic. This observation is supported by findings of other studies which shows that the prevalence of anaemia is higher among children under the age of five, with prevalence of up to 85% having been reported [43,52]. This observation highlights the need to target anaemia control interventions to younger children within and outside the school system with more emphasis on pre-school age children. Although many studies have reported a strong association between anaemia and malaria, in this study we did not find any relationship between the two, most likely because of the low prevalence of malaria in our study area.

One limitation of this study is the lack of dietary information for the study participants to be able to empirically associate the observed rates of undernutrition and anaemia with poor dietary nutrients.

## Conclusion

In conclusion, the current study has shown that *Schistosoma mansoni* infection, undernutrition and anaemia are highly prevalent in the study area. Although a number of studies have implicated *Schistosoma mansoni* infection as the cause of low haemoglobin levels and undernutrition, the present study failed to demonstrate this association among schoolchildren. This observation suggests that the observed higher levels of anaemia and undernutrition are likely to be a result of inadequate intake of essential dietary nutrients. We therefore recommend for policy makers to consider school age children in Rorya district for inclusion into national schistosomiasis control and school feeding programmes.

## Supporting Information

S1 Data S.mansoni, Malnutrition and Anaemia. (DTA)
